# Progress in Bacteriology

**Published:** 1903-12-12

**Authors:** 


### Progress in Bacteriology.

**General Paralysis of the Insane.**—Robertson[1] concludes that the changes in this disease are due to an active bacterial toxæmia. A general and local weakening of the body defences against bacteria takes place, there is an excessive development of various bacterial forms and a chronic toxic infection from the respiratory and alimentary tracts, the chief agent being a diphtheroid bacillus. The possibility of such an infection is brought about by the destruction of the leucoblastic function of the marrow by syphilis, alcohol, and other toxic substances. The bacillus found in general paralysis is an attenuated and saprophytic form of the Klebs Löffler bacillus which it closely resembles in cultural and morphological characteristics. Syphilis, by causing lesions in the upper respiratory and alimentary tract, provides a site for the absorption of the toxin, and it is noteworthy that disorders of digestion, chronic sore throat, septic conditions of the mouth, and chronic bronchitis are frequently found amongst general paralytics. In this disease, too, excessive numbers of bacteria are found in the alimentary canal after death, and during life cultures taken from the nose and throat swarm with bacteria. If the stomach contents be examined the same excess of saprophytic organisms is found and septic cystitis is common. The diphtheroid bacillus was obtained in 17 out of 20 cases, when cultures were made from the organs of general paralytics at post-mortem examinations. The same bacillus may be obtained if swabbings be taken from the throat and nose. It frequently takes part in a terminal infection and may be detected in the brain after death. A thread form of the diphtheria bacillus may frequently be observed in great numbers in the lymphatics of the alimentary tract, and is probably caused by the formation of anti-bodies. Robertson says that he has succeeded in growing these thread forms by cultivating the ordinary diphtheroid bacillus on the blood of general paralytics, or on that of an immunised rabbit. If rats be fed on broth containing cultures of this bacillus they develop changes in the nervous system comparable to those found in general paralysis, and these changes may terminate fatally. In the discussion which followed the paper Adami pointed out that chronic poisoning by B. coli was also capable of exciting in the nervous system of animals a progressive degenerative reaction.

**The Bactericidal Factors in Phototherapy.**—Whether bacteria in tissues such as lupus are destroyed by the direct effects of light or by the effects of the reactions set up by the light rays is at present uncertain. Barnard and Morgan[2] find that using a lamp with carbon electrodes and eliminating the effects of heat there is a powerful inhibiting effect upon the growth of colonies on the surface of agar plates, but that the deeper colonies are not affected. The fact that the bactericidal rays were devoid of penetrative power was further demonstrated by interposing between the lamp and the plates either layers of human skin or the web of living frog's foot; in both cases there was no interference with the growth of bacteria on the plates. The exact position of the bactericidal rays was found to be in about the middle third of the ultra violet, as seen in a photograph of the spectrum of carbon. No other rays were apparently active. The position of the rays which excite the reaction on the part of the tissues could not be determined, but there is evidence to show that they too are probably to be found in the ultra-violet band. Tested on hanging drop cultures of bacillus coli it was found that carbon electrodes charged with iron or cadmium have twice the bactericidal effect of ordinary carbons, and as the cadmium burns the most steadily this appears to be preferable to all the others. Finding that it took much longer to kill bacteria when suspended in water, an experiment was made to determine whether the water-cooled lens of an ordinary light absorbed bactericidal rays and this was proved to be the case. Light passing through 2 5 cm. of water lost four-fifths of its bactericidal power. The bactericidal power of a lamp is exactly proportionate to its light efficiency. These results lead us to the conclusion that the scientific treatment of disease by means of light is still in its infancy.

**Malaria.**—The following facts are summarised from the report to the Royal Society of Stephens and Christophers.[3] The evidence in favour of the theory of transmission by anopheles is undoubted. Blackwater fever is malarial in origin. Early in the disease parasites may be detected; later on this is often impossible, but pigmented leucocytes and the large meno-nuclear blood-count render the diagnosis certain. Blackwater fever is most commonly induced by quinine inefficiently administered. Many varieties of anopheles do not carry malaria in nature; this is true of A. Ressii and A. Barbirostris. A. Culicifacies and A. Christophersi carry the largest percentage of sperozoits. Quartan sperozoits were only found in A. Culicifacies. Each species of anopheles has its peculiar breeding place, A. Ressii in shallow, dirty pools; A. Barbirostris in weedy lakes; A. Christophersi in streams; A. Stephensi in tins. The flight of the mosquito rarely extends more than a half mile from the breeding ground.

Indian anopheles admit of a natural classification based upon the larval characters and those of the egg. Zygotes can develop in the unfertilised female anopheles.

**Sleeping Sickness.**[4]—Castellani recommends that in cases of sleeping sickness 15 c.c.s. of cerebro-spinal fluid be withdrawn. Rejecting the first 5 c.c.s., the remainder is centrifugalised and the sediment examined in the fresh state. In the fluid so examined trypanosomes were present in 70 per cent. of the cases. In 12 other cases of general disease, including three of trypanosome fever, no trypanosomes were found in the cerebro-spinal fluid. The trypanosoma found resembles that of Dutton (T. Gambiense), except that it is shorter, the micronucleus lies nearer the extremity, and the vacuole is apparently larger. Another point of difference is that the trypanosome of sleeping sickness moves with the flagellum behind. In 80 per cent. of the cases in which trypanosomes were found there was also present a streptococcus in cultures made from the fluid of the lateral ventricles of the brain and from the blood; this Castellani regards as a concomitant infection. Bruce and Nabarro[5] confirm in the main these observations. They found trypanosomes in the cerebro-spinal fluid of cases of sleeping sickness, not only in Uganda but in Kavirondo, and showed that they were present also in the blood in this disease. Though trypanosomes are present in the blood of a small percentage of natives they do not give rise to marked symptoms, and unless they enter the cerebro-spinal fluid they do not excite sleeping sickness. Inoculation experiments on monkeys do not afford any evidence at present as to the identity or otherwise of the trypanosomes found in cases of sleeping sickness and those found in trypanosoma fever. Sleeping sickness occurs almost solely in Uganda along the northern shores of Victoria Nyanza in a strip of country about fifteen miles wide. The trypanosomes are probably transmitted to men and animals by the bite of the tsetse fly (Glossina palpalis), and this has been experimentally verified on the monkey. The distribution of sleeping sickness in East Equatorial Africa has been determined by Christy,[6] and coincides with that mentioned above. Castellani[7] gives the following details of adult and developmental forms of the trypanosome found in sleeping sickness. It is of a worm-like shape, one end blunted, the other terminating in a flagellum. The body has an undulating membrane and a vacuole. The protoplasm has an alveolar structure. Watching fresh specimens one may see occasionally the parasite engulfed by a leucocyte or suddenly diappear as if dissolved. When on the point of dividing two flagella can sometimes be seen. Parasites may be kept alive in blood serum for from four to six hours, in cerebro-spinal fluid from 15 to 18 hours. They have not been found in the tissues. When preparing for division the trypanosome becomes altered in shape, thicker, rounded, more vacuolated. Others appear as Rabinovitsh-Kempner bodies and Plimmer-Bradford amœboid forms. In the same report Low disposes of the theory that the disease is in any way connected with the presence of *Filaria perstans*, and the same author, in conjunction with Castellani, deals in considerable detail with the clinical aspects of sleeping sickness.

**Micro-organisms in the Air.**—Graham Smith[8] has made an investigation of the air of the House of Commons. The method pursued was that of aspirating a known quantity of air through a sterile glass tube in which were plugs of finely-divided glass wool. Micro-organisms were caught in the plugs, and at the conclusion of the experiment the tube was cut up and the plugs dropped into melted sterile gelatin, and the resulting colonies counted. He found that the air in the open space surrounding the Houses of Parliament there were very few bacteria (4·2 per litre), and at the top of the clock tower only about one-third of the number. In the debating chamber itself, during a sitting, the number only reached 5·8 per litre, but in the committee, dining, and smoking-rooms the average was 32·3 per litre. No organisms associated with specific diseases in man were isolated, and only a few pathogenic to animals. These investigations were undertaken in the summer of 1902, and Frankland and others have shown that, starting from a minimum in January, the number of organisms in the air reaches a maximum in August, and then declines. Graham Smith isolated 20 species of bacilli, and 27 of cocci from the air of the House of Commons.

[1] Brit. Med. Jour., Oct. 24, 1903. [2] Brit. Med. Jour., Nov. 14, 1903. [3] Report of Malaria Com., Oct. 10, 1903. [4] Royal Soc. Rep. of Sleeping Sickness Com., No. 1, Aug., 1903. [5] Ibid. [6] Royal Soc. Rep. of Sleeping Sickness Com., No. 2, Nov., 1903. [7] Ibid. [8] Jour. of Hygiene, Oct., 1903.

